# *Candida albicans* Impairments Induced by Peppermint and Clove Oils at Sub-Inhibitory Concentrations

**DOI:** 10.3390/ijms18061307

**Published:** 2017-06-19

**Authors:** Katarzyna Rajkowska, Anna Otlewska, Alina Kunicka-Styczyńska, Agnieszka Krajewska

**Affiliations:** 1 Institute of Fermentation Technology and Microbiology, Faculty of Biotechnology and Food Sciences, Lodz University of Technology, Lodz 90-924, Poland; anna.otlewska@p.lodz.pl (A.O.); alina.kunicka@p.lodz.pl (A.K.-S.); 2 Institute of General Food Chemistry, Faculty of Biotechnology and Food Sciences, Lodz University of Technology, Lodz 90-924, Poland; agnieszka.krajewska@p.lodz.pl

**Keywords:** *Candida albicans*, essential oils, anticandidal activity, enzymatic activity, karyotype, proteins profile

## Abstract

Members of *Candida* species cause significant health problems, inducing various types of superficial and deep-seated mycoses in humans. In order to prevent from *Candida* sp. development, essential oils are more and more frequently applied, due to their antifungal activity, low toxicity if used appropriately, and biodegrability. The aim of the study was to characterize the early alterations in *Candida albicans* metabolic properties in relation to proteins and chromosomal DNA profiles, after treatment with peppermint and clove oils at sub-inhibitory concentrations. The yeasts were affected by the oils even at a concentration of 0.0075% *v*/*v*, which resulted in changes in colony morphotypes and metabolic activities. Peppermint and clove oils at concentrations ranging from 0.015× MIC (minimal inhibitory concentration) to 0.5× MIC values substantially affected the enzymatic abilities of *C. albicans*, and these changes were primarily associated with the loss or decrease of activity of all 9 enzymes detected in the untreated yeast. Moreover, 29% isolates showed additional activity of *N*-acetyl-β-glucosaminidase and 14% isolates—α-fucosidase in comparison to the yeast grown without essential oils addition. In response to essential oils at 0.25–0.5× MIC, extensive changes in *C. albicans* whole-cell protein profiles were noted. However, the yeast biochemical profiles were intact with the sole exception of the isolate treated with clove oil at 0.5× MIC. The alterations were not attributed to gross chromosomal rearrangements in *C. albicans* karyotype. The predominantly observed decrease in protein fractions and the yeast enzymatic activity after treatment with the oils should be considered as a phenotypic response of *C. albicans* to the essential oils at their sub-inhibitory concentrations and may lead to the reduction of this yeast pathogenicity.

## 1. Introduction

*Candida* species are currently the most common cause of fungal infections worldwide [1], and a multicenter candidemia study revealed *Candida albicans* to be the most frequent fungal pathogen [2]. Among factors contributing to the pathogenic potential of *C. albicans* is the production of adhesins and invasins, which mediate adhesion to and invasion of host cells, the secretion of hydrolytic enzymes, the yeast-to-hypha transition, contact sensing and thigmotropism, biofilm formation, phenotypic switching, and metabolic adaptability [3]. Due to increased resistance and cross-resistance to antibiotics of *C. albicans* isolates and the 25–60% mortality of patients with candidemia [4,5], there is growing interest in the use of natural products derived from medicinal plants as antifungal agents. Essential oils (EOs) have already found a considerable range of applications because of their broad spectrum of activities, fewer side effects, lower toxicity if used appropriately, and superior biodegrability compared with chemicals [6,7,8]. However, their usage in therapy may be limited because of irritant and contact allergy reactions. Toxicity of essential oils depends distinctly on their concentration, composition, and route of administration, and adverse reactions can be avoided through the use of lower concentrations of EOs [9].

Peppermint and clove oils and their major components are well known for their antifungal activity [10,11]. This antimicrobial action of essential oils is primarily explained by their cytotoxic effect due to damage and changes in the fluidity of membranes [10]. Scanning and transmission electron microscopy reveal significant morphological alterations with cellular deformity of yeast cells in the presence of essential oils [10,12].

The researchers have mainly focused on the antimicrobial activity and mechanism of action of essential oils applied at lethal concentrations. Previously, we demonstrated the broad spectrum of changes in *C. albicans* morphology and metabolic activities, induced by thyme and tea tree oils at sub-inhibitory concentrations [13]. The aim of this study was to determine if the phenomenon of morphological and metabolic changes of yeast at essential oil concentrations far below their lethal ranges is a universal mechanism, appropriate also for other oils. From a variety of EOs, peppermint (*Mentha piperita* L.) and clove (*Syzygium aromaticum* (L.) Merr. & L.M. Perry) oils, EOs of distinct chemical compositions from tea tree and thyme oils, were chosen. In the context of changes in yeast metabolic activities, we also set out to determine whether EOs at sub-inhibitory concentrations can cause alterations in yeast proteins and chromosomal DNA profiles. The recognition of early alterations in the cells’ behaviour in response to essential oils may contribute to an explanation of the EOs’ mechanisms of action on *C. albicans*.

## 2. Results and Discussion

The growth of *C. albicans* was observed on solid medium supplemented with peppermint and clove oils at concentrations ranging from 0.0075 to 0.25% *v*/*v*, corresponding to half or less of their minimum inhibitory concentrations (MIC of peppermint oil: 1.0% *v*/*v*, MIC of clove oil: 0.5% *v*/*v*, determined in our previous study) [14]. Irrespective of the type and concentration of the essential oil, morphological changes of the colonies were noted. Out of 63 colonies 7 colonies representing different altered morphotypes were chosen (Table 1).

According to the published yeast colony morphotypes [15], irregular (PO/0.25, CLO/0.0075) and spokes (PO/0.015) colonies were obtained. Following the treatment with peppermint and clove oils the other colonies observed were smooth, but differed in size. The smallest colonies were detected in the presence of peppermint oil at concentrations of 0.0075% and 0.25%, and clove oil at 0.125% *v*/*v*. The formation of petite colonies in *C. albicans* is typical for mutants deficient in mitochondrial respiration, explained by their lower cell division rates than for normal cells and a decrease in biomass yield [16]. Moreover, as shown previously, the terpene components of essential oils at lower concentrations could inhibit yeast respiration and, at higher concentrations, could increase the permeability of the outer and inner mitochondrial membranes [17]. The presence of the tiny colonies, indicating alterations in *C. albicans* mitochondrial function, are consistent with our previous results of mitochondrial damage in eukaryotic cells [18].

The altered *Candida* morphotypes may be caused by numerous environmental factors, including fluctuations in temperature and pH, nutrient levels, and serum exposure [19]. Alterations of *C. albicans* colony morphology might be associated with chromosomal DNA rearrangements [20]. To check the impact of peppermint and clove oils at sub-inhibitory concentrations on the yeast karyotype, electrophoretic analysis of *C. albicans* chromosomal DNA was performed.

**Figure 1:**
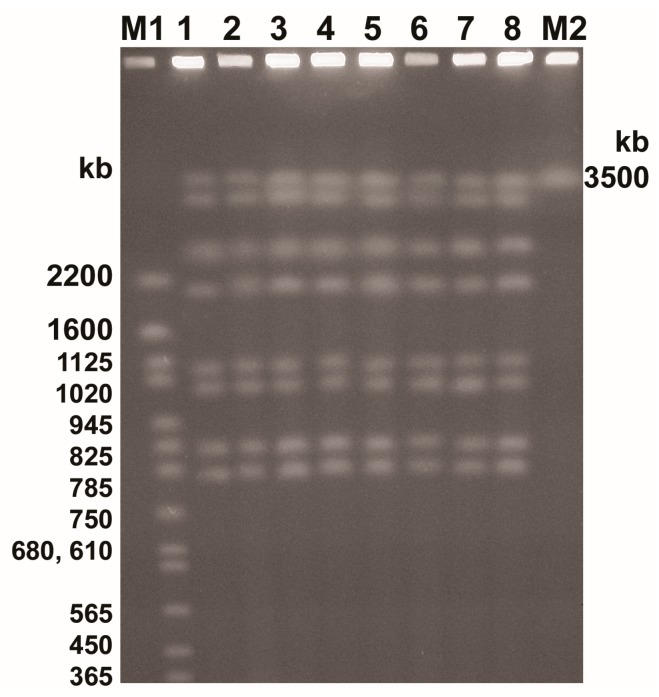
Electrophoretic karyotypes of different *Candida albicans* ATCC 10231 morphotypes, after treatment with peppermint oil (PO) and clove oil (CLO). **M1**—DNA size marker *Saccharomyces cerevisiae* YNN295 (Bio-Rad); **1**—control; **2**—PO/0.25; **3**—PO/0.015; **4**—PO/0.0075/1; **5**—PO/0.0075/2; **6**—CLO/0.25; **7**—CLO/0.125; **8**—CLO/0.0075; **M2**–DNA size marker *Schizosaccharomyces pombe* 972h-(Bio-Rad). Acronyms are composed as follows: essential oil type/oil concentration/isolate number (if any).

The chromosomal DNA of *C. albicans* ATCCC 10231 was separated into 8 bands ranging in size from 780 to 3570 kb (Figure 1), which is consistent with previously reported results for clinical *C. albicans* strains [21]. After treatment with peppermint and clove oils, irrespective of their concentration, no changes in yeasts electrophoretic karyotypes were observed (Figure 1). Therefore, the morphological changes observed in this study in response to essential oils were not attributed to gross modifications of chromosomal DNA. It is accepted that adaptive mutations in *C. albicans*, generating diversity in response to stressful growth conditions (including antifungal drug exposure) occur through point mutations, recombination, gene conversion, loss of heterozygosity, and/or aneuploidy [20]. Although these types of genomic changes after treatment with essential oils cannot be excluded, it has also been reported that EOs did not induce point mutations or mitotic intragenic or intergenic recombination [22].

In this context, the extensive changes in enzymatic activity and the slight differences in biochemical profiles of *C. albicans* in the presence of the investigated essential oils should be instead considered as phenotypic response to the EOs. *C. albicans* grown without essential oils addition showed the ability to utilise glucose, 2-keto-d-gluconate, xylose, adonitol, xylitol, galactose, sorbitol, methyl-αd-glucopyranoside, *N*-acetyl-glucosamine, maltose, saccharose, and trehalose. The only isolate *C. albicans* CLO/0.25 lost the ability to assimilate xylitol after treatment with 0.25% clove oil. For pathogenic fungi, the effective assimilation of various nutrients is particularly important for enabling the colonization of different host niches [23]. The tested essential oils at the concentrations below their MIC values practically do not limit *C. albicans* ability to utilize different carbon sources.

**Figure 2:**
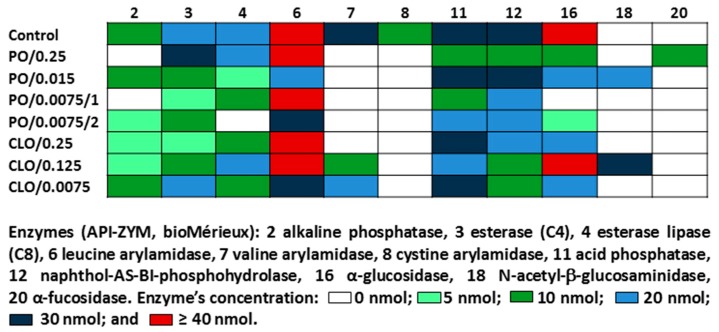
*Candida albicans* ATCC 10231 enzymatic profiles after treatment with peppermint oil (PO) and clove oil (CLO). Acronyms are composed as follows: essential oil type/ oil concentration/ isolate number (if any).

Changes in the enzymatic profiles of *C. albicans* isolates were primarily associated with a loss or decrease in the activity of all the enzymes detected for yeast not treated with essential oils, i.e., alkaline phosphatase, esterase, esterase lipase, leucine arylamidase, valine arylamidase, cystine arylamidase, acid phosphatase, naphthol-AS-BI-phosphohydrolase, and α-glucosidase (Figure 2). However, for the isolates PO/0.015 (0.015% peppermint oil treatment) and CLO/0.125 (0.125% clove oil treatment), an *N*-acetyl-β-glucosaminidase activity was detected, but not observed for *C. albicans* grown without oils. Moreover, the isolate PO/0.25 (0.25% peppermint oil treatment) showed an additional hydrolytic activity of α-fucosidase and an increase in esterase activity.

The actions of *Candida* extracellular enzymes may contribute as virulence factors for the establishment of infection by providing access to nutrients or affecting the function and viability of the host [24]. Hence, the observed decrease in enzymatic activity of *C. albicans* isolates could significantly reduce their pathogenicity. Phospholipases and proteases play a key role in the pathogenesis of *Candida* spp. [25]. Phospholipases hydrolyze the ester linkages of glycophospholipids and impart tissue invasiveness to *Candida* cells [24,25]. Proteases may cause the degradation of epithelial cells, cytokines, and immunoglobulins and the modification of fungal surface antigens and thus contribute to the process of adhesion and colonization of host tissues [25,26]. Secreted aspartyl proteinases have been reported to play a complex role in the pathogenicity of *C. albicans*, and their production is also correlated with hyphal formation, adherence, and phenotypic switching [27]. Furthermore, alkaline phosphatase, *N*-acetyl-β-glucosaminidase, and α-mannosidase inhibit the migration of neutrophils to the sites of infection, and lipase enables the use of lipids as a carbon source by fungi and allows their further growth and the development of infection [24]. The acquired ability of two isolates to produce *N*-acetyl-β-glucosaminidase and one isolate to secrete α-fucosidase is a change, suggesting the defensive yeast answer to peppermint and clove oils at concentrations far below their lethal values. On the other hand, the observed changes in the hydrolytic enzymes *N*-acetyl-β-glucosaminidase and α-fucosidase may give important new hints to counteract the progression of *C. albicans* infections by therapeutic interference with these enzymes.

**Figure 3:**
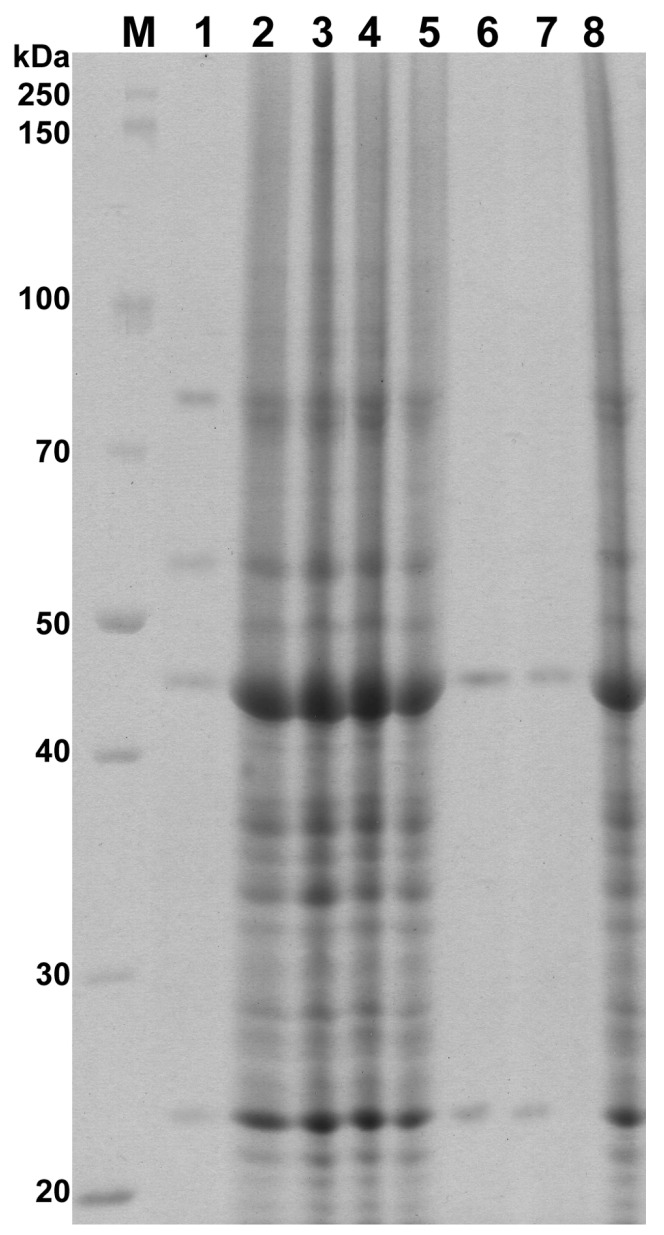
Protein profiles of different *Candida albicans* ATCC 10231 morphotypes, after treatment with peppermint oil (PO) and clove oil (CLO); **M**—marker (PageRuler Broad Range Unstained Protein Ladder, Thermo Scientific); **1**—PO/0.25; **2**—PO/0.015; **3**—PO/0.0075/1; **4**—PO/0.0075/2; **5**—control; **6**—CLO/0.25; **7**—CLO/0.125; **8**—CLO/0.0075. Acronyms are composed as follows: essential oil type/oil concentration/isolate number (if any).

In our study, *C. albicans* colony morphology was linked to changes in protein profiles, but only after treatment with oils at the concentrations 0.125–0.25% *v*/*v*. Analysis of the whole cell proteins of *C. albicans* demonstrated 28 protein fractions with molecular weights ranging between 19 and 110 kDa (Figure 3), which is consistent with previously published results [28].

The number of protein fractions in the profiles of PO/0.25, CLO/0.25, and CLO/0.125 isolates obtained after treatment with 0.25% peppermint, 0.25% clove, and 0.125% clove oils, respectively, was distinctly reduced in comparison with the profile of the strain not subjected to the essential oils (Figure 3). In *C. albicans* protein profiles after treatment with 0.25% peppermint oil, only four proteins of the molecular weights 80, 56, 44, and 23 kDa were noticed (Figure 3, Line 1). However, in response to 0.25% and 0.125% clove oil, the two proteins 44 and 23 kDa were merely observed (Figure 3, Lines 6 and 7).

The level of isolates profiles similarity, counted as Dice index, equalled 25.0% for PO/0.25 (0.25% peppermint oil treatment) and 13.3% for CLO/0.25 and CLO/0.125 (clove oil treatment at 0.25 and 0.125%, respectively) as compared to the untreated yeasts (Table 2).

Analysis of electrophoretic profiles of proteins has been satisfactorily employed in the characterization of yeasts, and in the identification, classification, and even in the reclassification of numerous strains, species, and genera of microorganisms in taxonomic and epidemiological studies [28,29,30]. In addition, variations in this technique made it possible to analyze the intra- and inter-specific variability of *Candida* spp. [30]. Such a broad application of this technique suggests that protein profiles obtained by SDS-PAGE provide relatively stable taxonomic characteristics. The decrease in the number of fractions in whole cell proteins profiles of *C. albicans* can be explained by the loss of cell membrane integrity and leakage of intracellular compounds induced by essential oils, as we have shown previously [18].

The action of peppermint and clove essential oils is considered to be related to their active components, i.e., eugenol, β-caryophyllene, menthol, and menthone [6,10,11]. The major constituents of clove oil used in our study were eugenol and (*E*)-β-caryophyllene, which represented 95.1% of this oil total composition (Table 3). In peppermint oil, a total of 24 compounds were identified, with predominant menthol (43.9%) and menthone (23.1%). A high concentration of these active compounds in the tested essential oils may be the explanation of their anticandidal activity. The antimicrobial activity of EOs and their monoterpenoid components is primarily explained by their toxic effects on membranes structure and function [7,10,18]. It has been reported that the exposure to essential oils and their components causes the release of intracellular compounds, including radicals, cytochrome C, calcium, potassium, and magnesium ions, proteins, and nucleic acids, leading to cell death [10,31]. On the other hand, the effects may also be due to the oil-induced production of radicals and oxidative stress [32]. Cytotoxic and genotoxic activity were already recognized for essential oils [6,17,18,32], but an explanation of mechanisms of action of peppermint and clove oils on yeast proteome requires further studies.

## 3. Materials and Methods

### 3.1. Yeast

The study was carried out for *C. albicans* ATCC 10231, commonly used as a reference strain in the analysis of antifungal agents. The strain was maintained on Sabouraud dextrose agar slants (5 g/L peptone from meat, 5 g/L peptone from casein, 20 g/L dextrose, and 20 g/L agar) and activated by subculturing in Sabouraud broth at 37 °C for 24 h.

### 3.2. Essential Oils

Essential oils from *Mentha piperita* L. (peppermint oil) and *Syzygium aromaticum* (L.) Merr. & L.M. Perry (clove oil) were obtained from Pollena Aroma S.A., Warsaw, Poland.

The chemical compositions of the essential oils were analyzed by gas chromatography mass spectrometry flame ionization detection (GC-MS-FID) using a Trace GC Ultra (Thermo Scientific, Waltham, USA) chromatograph combined with a DSQ II mass spectrometer with a flame ionization detector (FID) using an MS-FID splitter (SGE, Analytical Science, Trajan, Australia) and a nonpolar capillary column, Rtx-1 ms (60 m × 0.25 mm, film thickness 0.25 μm, Restek, Bellefonte, PA, USA). The oven temperature was programmed as followed: 50–300 °C at 4 °C/min; injector temp.: 280 °C; detector temp.: 310 °C; carrier gas helium with regular pressure: 200 kPa; ionization energy: 70 eV; ion source temperature: 200 °C. Components were identified based on comparisons of their mass spectra with those of a laboratory-made MS library and commercial libraries (Adams [33], NIST 09, Wiley 275.1 and Mass Finder 4) and with retention indices associated with a series of alkanes using linear interpolation (C8–C26). Quantitative analyses (expressed as percentages of each component) were carried out using peak area-normalized measurements without correction factors. The identified essential oil components are presented in Table 3.

### 3.3. Colony Morphology Assay

A *C. albicans* cell suspension was prepared in sterile saline (8.5 g/L sodium chloride) from a 24-hour-old culture grown on Sabouraud agar medium at 37 °C. Yeast cells were counted with a hemocytometer, and serial 10-fold dilutions were then performed to obtain approximately 300 cells in 1 mL of saline. One hundred microliters of this solution was streaked onto Sabouraud dextrose agar with the addition of peppermint or clove oil at a concentration of 0.0075, 0.015, 0.03, 0.06, 0.125, 0.25, 0.5, and 1.0% *v*/*v* in an agar absorption assay [34]. As a surfactant 0.2% Tween 80 (polisorbate 80) was used. The control was *C. albicans* grown on Sabouraud dextrose agar with the addition of Tween 80. Plates were incubated at 37 °C for up to 14 days. Yeast colonies of different morphologies were transferred to 5 mL of sterile saline and used for further studies.

### 3.4. Biochemical and Enzymatic Profiles

The biochemical ability of the *C. albicans* reference strain and isolates to assimilate 19 substrates (d-glucose, glycerol, 2-keto-d-gluconate, l-arabinose, d-xylose, adonitol, xylitol, d-galactose, inositol, d-sorbitol, methyl-αd-glucopyranoside, *N*-acetyl-glucosamine, d-cellobiose, d-lactose, d-maltose, d-saccharose, d-trehalose, d-melezitose, and d-raffinose) as the sole carbon sources was checked with API 20C AUX tests (bioMérieux, Marcy l’Etoile, France) according to the manufacturer’s guidelines.

To determine enzymatic profiles of yeasts, the API-ZYM test (bioMérieux) was used, and the activities of alkaline phosphatase, esterase (C4), esterase lipase (C8), lipase (C14), leucine arylamidase, valine arylamidase, cystine arylamidase, trypsin, α-chymotrypsin, acid phosphatase, naphthol-AS-BI-phosphohydrolase, α-galactosidase, β-galactosidase, β-glucuronidase, α-glucosidase, β-glucosidase, *N*-acetyl-β-glucosaminidase, α-mannosidase, and α-fucosidase were checked.

### 3.5. Protein Preparation and Electrophoresis

Whole cell proteins of samples were extracted according to Duran et al. [35] with some modifications. Sabouraud dextrose agar with the addition of essential oils at the appropriate concentration was prepared. Suspensions of each yeast colony of different morphology were streaked onto an agar medium, subject to the type and concentration of essential oil, similarly as with isolation of specific morphotypes. After incubation at 37 °C for 24 h, two loopfuls of yeast biomass were collected from the surfaces of plates and transferred to 10 mL of sterile saline. For protein preparation, 5 mL of yeast cells solutions were harvested by centrifugation at 4000 rpm for 5 min and washed twice with distilled water. Pellets suspended in 0.5 mL of saline were combined with 5 µL of PMSF (phenylmethylsulfonyl fluoride 0.1 M), 5 µL of 2-mercaptoethanol (10%), and 5 µL of EDTA (ethylenediaminetetraacetic acid 1.0 M) and homogenized with a sonicater. After centrifugation (10,000 rpm, 10 min), 300 µL of supernatants were collected and incubated with 300 µL of ethanol at −20 °C for 30 min. To the pellets, 25 µL of sample buffer (0.06 M Tris-HCl pH 6.8, 10% glycerol, 2% sodium dodecyl sulphate, 5% 2-mercaptoethanol, and 0.025% bromophenol blue) were added and heated in a boiling water bath for 5 min. The protein concentration was determined by NanoPhotometer™ Pearl UV-Vis spectrophotometer (Implen GmbH) according to the manufacturer’s instructions.

Sodium dodecyl sulphate polyacrylamide gel electrophoresis (SDS-PAGE) of proteins was performed according to the method of Laemmli [36], using 5% stacking gel and 10% separating gel in TGS buffer (25 mM Tris-HCl pH 8.6, 192 mM glycine, and 0.1% SDS) at 30 mA. Proteins in the gel were stained with Coomassie Brillant Blue R-250 (0.25% Coomassie Brillant Blue R-250, 10% acetic acid, 45% methanol).

### 3.6. Chromosomal DNA Analysis

Chromosomal DNA was isolated from the remaining 5 mL of yeast cells solutions, prepared as described in protein preparation. DNA isolation was conducted using a CHEF Genomic DNA Plug kit (Bio-Rad, Hercules, CA, USA) according to the methods described by Schwartz and Cantor [37]. Chromosomes were separated by pulsed field gel electrophoresis (PFGE) in 0.8% agarose gel by means of a CHEF-DR II apparatus (Bio-Rad). Electrophoresis was performed in 0.5× TBE buffer (45 mM Tris, 45 mM boric acid, and 10 mM EDTA; pH 8.2) at 12 °C, and the pulses were as follows: 120 s for 24 h and 240 to 360 s for 24 h, all at 4.5 V/cm. Separated chromosomes were stained in ethidium bromide (0.5 µg/mL) for 15 min with gentle agitation. The molecular weight of bands was estimated using SigmaGel software (Sigma-Aldrich, Gillingham, UK).

### 3.7. Statistical Analysis

All experiments were carried out in 3 independent experiments. To compare protein profiles in SDS-PAGE, the Dice index of similarity coefficient was calculated using the formula:

*S* (%) = 2*X* × 100/(*a* + *b*) (1)

where *a* is the total number of bands of the control, *b* is the total number of bands of the isolate, and *X* is the total number of similar bands in both lanes [28].

## 4. Conclusions

The changes observed for *C. albicans* in the presence of peppermint and clove oils, especially in yeast enzymatic activity and protein profiles, may contribute to the reduction of *C. albicans* pathogenicity. This finding is consistent with our previous report for thyme and tea tree oils [13]. The comparable action of essential oils of different chemical compositions indicates that this mechanism may be universal for EOs, and their use, even at below their minimum inhibitory concentrations, may limit enzymatic activity of *C. albicans*. To assess the potential of the tested essential oils and their influence on the yeast proteome, further research will be undertaken.

## Figures and Tables

**Table 1:** Colony morphotypes of *Candida albicans* ATCC 10231 in the presence of peppermint oil (PO) and clove oil (CLO). Acronyms are composed as follows: essential oil type/oil concentration/isolate number (if any).

Essential Oil	Oil Concentration (% *v*/*v*)	The Number of Colonies with Different Morphology/the Total Number of Colonies on the Plate ^1^	Representative Colony with Different Morphology	Isolate Acronym
Peppermint oil	0.25	1/1	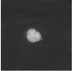	PO/0.25
	0.015	11/19	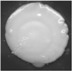	PO/0.015
	0.0075	10/26	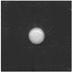	PO/0.0075/1
		13/26	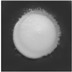	PO/0.0075/2
Clove oil	0.25	4/5	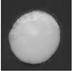	CLO/0.25
	0.125	1/1	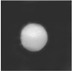	CLO/0.125
	0.0075	9/11	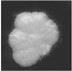	CLO/0.0075
Without essential oil	—	0/28	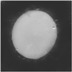	Control

^1^ Data presented only for these variants with colonies of different morphology.

**Table 2:** Similarity of protein profiles of *Candida albicans* ATCC 10231 isolates, after treatment with peppermint oil (PO) and clove oil (CLO). Acronyms are composed as follows: essential oil type/oil concentration/isolate number (if any).

Isolate	Whole Cell Proteins (kDa)	Dice Index of Similarity (%)
Control	110, 95, 89, 80, 75, 65, 56, 49, 44, 40, 39, 38, 36, 35, 34, 33, 32, 31, 30, 28, 27, 26, 25, 24, 23, 21, 20, 19	—
PO/0.25	80, 56, 44, 23	25.0
PO/0.015	110, 95, 89, 80, 75, 65, 56, 49, 44, 40, 39, 38, 36, 35, 34, 33, 32, 31, 30, 28, 27, 26, 25, 24, 23, 21, 20, 19	100.0
PO/0.0075/1	110, 95, 89, 80, 75, 65, 56, 49, 44, 40, 39, 38, 36, 35, 34, 33, 32, 31, 30, 28, 27, 26, 25, 24, 23, 21, 20, 19	100.0
PO/0.0075/2	110, 95, 89, 80, 75, 65, 56, 49, 44, 40, 39, 38, 36, 35, 34, 33, 32, 31, 30, 28, 27, 26, 25, 24, 23, 21, 20, 19	100.0
CLO/0.25	44, 23	13.3
CLO/0.125	44, 23	13.3
CLO/0.0075	110, 95, 89, 80, 75, 65, 56, 49, 44, 40, 39, 38, 36, 35, 34, 33, 32, 31, 30, 28, 27, 26, 25, 24, 23, 21, 20, 19	100.0

**Table 3:** Composition of essential oils (GC-MS analysis); RI: retention index, —: not detected.

Compound	RI	Peppermint Oil	Clove oil
		Content (%)	
α-Pinene	934	0.6	–
Sabinene	968	0.2	–
β-Pinene	974	1.0	–
*p*-Cymene	1016	0.3	–
1,8-Cineole	1020	6.6	–
Limonene	1025	2.4	–
*trans*-Sabinene hydrate	1060	0.2	–
2-Methylbutyl 2-methylbutanoate	1094	0.1	–
Menthone	1138	23.1	–
Isomenthone	1145	3.8	–
Menthofuran	1154	2.2	–
Neomenthol	1155	3.3	–
Menthol	1163	43.9	–
Neoisomenthol	1173	1.0	–
α-Terpineol	1178	0.6	–
Isomenthol	1179	0.2	–
Pulegone	1218	1.2	–
Piperitone	1237	0.4	–
Menthyl acetate	1279	4.9	–
Eugenol	1342	–	85.2
β-Burbonene	1381	0.1	–
Methyleugenol	1386	–	0.2
(*E*)-β-Caryophyllene	1421	1.6	9.9
α-Humulene	1453	0.1	1.9
Germacrene D	1474	0.3	–
δ-Cadinene	1513	–	0.4
(*E*)-β-Caryophyllene oxide	1573	0.2	0.4

## Abbreviations

EO	Essential oil
PO	Peppermint oil
CLO	Clove oil
*C. albicans*	*Candida albicans*
MIC	Minimal inhibitory concentration
